# Peer-to-Peer Social Media as an Effective Prevention Strategy: Quasi-Experimental Evaluation

**DOI:** 10.2196/16207

**Published:** 2020-05-06

**Authors:** William Evans, Elizabeth Andrade, Michaela Pratt, Alexandra Mottern, Sergio Chavez, Anthony Calzetta-Raymond, Jiayan Gu

**Affiliations:** 1 Milken Institute School of Public Health George Washington University Washington, DC United States; 2 Mentor Foundation USA Tysons, VA United States

**Keywords:** social media, substance use, prevention, marijuana, opioids, adolescent health

## Abstract

**Background:**

Substance use by adolescents remains to be at unacceptably high levels, and there is evidence that teens’ social norms are becoming more favorable toward recreational use and perceived safety of substances such as marijuana and prescription opioids. Social media offer a low-cost, potentially high-impact approach to disseminate prevention messages.

**Objective:**

Living the Example (LTE) is a program that trains adolescent youth ambassadors to develop and disseminate prevention messages within their own social media networks and through in-school activities. This study aimed to evaluate the effects of exposure to LTE-based social media on students in the youth ambassadors’ networks.

**Methods:**

The George Washington (GW) University designed and implemented a quasi-experimental evaluation of the LTE program in 3 Maryland high schools. Before program launch, a sample of 826 students (wave 1) at the 3 schools, drawn from a census of freshmen enrolled in a class attended by all students at the grade level, completed a survey. A total of 584 students were surveyed at the wave 2 program midpoint and 542 at the wave 3 endpoint. The survey contained questions on drug use–related attitudes, beliefs, intentions, and behaviors, all based on validated measures. We evaluated the effects of LTE on the intended next 30-day drug use, and controlling for LTE self-reported exposure, age, and gender from waves 2 and 3 was appended into a single dataset. We first conducted ordinal logistic regressions for each drug use intention in wave 3 (ie, sell or distribute illegal drugs, smoke cigarettes, drink beer/wine/hard liquor when parents do not know about it, use marijuana, use lysergic acid diethylamide, cocaine, amphetamines or other illegal drugs, use heroin, use synthetic drugs, and use any prescription pills without a prescription) to examine the association between LTE exposure and drug use intentions. We included an interaction term for the study wave to examine intervention effects.

**Results:**

We found a significant positive effect of LTE exposure on all 8 measured drug use intentions: sell/distribute illegal drugs; smoke cigarettes; drink beer, wine, or liquor when my parents do not know about it; use marijuana; use cocaine, amphetamines, or other illegal drug; use heroin; use synthetic drugs; use any prescription pills without a prescription (all *P*<.05; odds ratios ranging from 2.12 to 3.71). We also found that boys were more likely than girls to exhibit reduced drug use intentions. We also found reductions in 30-day intentions between the second and third survey waves for all 8 measured drug use variables.

**Conclusions:**

Overall, the results are consistent with and indicate a stronger LTE effect in this study compared with a previous pilot study. LTE appears to offer a protective effect, with exposure to program messages leading to reduced/improved drug use intentions.

## Introduction

### Background

As shown in the most recent Monitoring the Future study, substance abuse is among the greatest public health threats [1]. Substance use by adolescents remains to be at unacceptably high levels, and there is evidence that teens’ social norms are becoming more favorable toward recreational use and perceived relative safety of substances such as marijuana and prescription opioids [2-4]. Teens are particularly sensitive to the messaging about drugs in their social environment, including in digital communities, and drug use choices are driven by availability, acceptability, and perceived risk [5,6]. Recently there have been many changes in the availability of marijuana to the public. The consequences of use and the situations in which individuals may choose to use marijuana are changing in many states owing to medical marijuana and legalization [7], potentially influencing youth perceptions of acceptability and risk [8]. Furthermore, due to widespread availability and a landscape of more dangerous drugs, including fentanyl contamination, opioid overdose death rates continue to rise despite overall declines in use [9,10]. Yet, some US $40 billion per year is spent on prevention programs [11], including major efforts involving health communication, such as the Above the Influence campaign [12]. Given this growing public health threat, prevention efforts must prioritize engagement strategies that address the evolving substance use risk perceptions and leverage youths’ and young adults’ affinity for digital technology [13].

There is growing evidence that marijuana use has negative health consequences for adolescents, especially when use begins early and when combined with other substance use [14]. Recent studies suggest adolescent marijuana use may be linked to altered longer-term neurodevelopmental trajectories and compromised neural health, impaired frontal lobe function, and psychosocial effects [14-16]. Additionally, early-onset adolescent marijuana use combined with alcohol and other substance use has been linked to numerous cognitive impairments and neural health effects [17,18]. Social norms favoring marijuana use are increasing [19,20] and may be associated with the relaxation of marijuana laws [21,22]. At the same time, both natural and synthetic opioid use has clear and well-documented health consequences, and adolescents are at a heightened risk for long-term neurodevelopmental effects [23]. Approximately 68% of the 70,237 drug overdose deaths in 2017 involved opioids, and over 28,466 of those deaths were from synthetic opioids such as fentanyl [24].

### Social Media and Prevention

Social media and social networks are major drivers of public debate and perceptions of marijuana, opioids, and other drugs [7,14,15,25,26], including for the youth, and there are numerous messages promoting the acceptability of substance use in these digital networks. This highlights the need for novel interventions that use digital prevention strategies and offer a counternarrative or counterargument [27]. Recent studies have examined exposure to antidrug communications and found that counternarrative strategies may partially explain positive campaign outcomes [28,29]. A campaign’s *brand marketing strategy* may in part determine how the audience responds to antidrug advertising [12]. Substance abuse prevention programs and behaviors promoted by these programs can have brand identities as well. Recent studies have linked drug resistance branding to improved substance use outcomes [30,31]. Our research team has pioneered branding for prevention in multiple domains [32,33]. In the context of relaxed marijuana laws and heightened opioid risk, this study investigated how prevention can be optimized through branded peer-to-peer messaging and digital engagement.

The Mentor Foundation USA implements a program entitled, Shatter the Myths (STM) Youth Rallies—this program conducts interactive in-person events with high school students and is designed to raise an awareness of risks and dispel myths surrounding drug use [34-37]. STM Youth Rallies are based on recent neuroscience, use an experiential learning approach to prevention, and apply principles from branding and social marketing [38,39]. By focusing on youths’ innate talents and strengths, the rallies enable youths to become advocates regarding the benefits of living a drug-free lifestyle.

Mentor Foundation USA is part of the Mentor International organization, which implements youth development programs worldwide. Mentor International’s work is guided by the Positive Youth Development (PYD) theory [40], working with children and families to combat risk behaviors and promote healthy lifestyles. Mentor International follows the United Nations Sustainable Development Goals, in particular, by ensuring healthy lives and promoting well-being for all ages.

Living the Example (LTE) has been designed based on the PYD theory, following the authors’ previous research, to address the rapidly changing substance use social environment in the United States by empowering the youth to serve as peer change agents [41,42]. LTE builds upon the STM Youth Rallies by adding a social media–delivered component to spread peer-to-peer substance use prevention messages, while also applying social marketing and branding approaches.

LTE represents a novel approach to addressing a wide range of substances used by adolescents through gain-framed messaging and digital engagement. According to the Prospect Theory, framing warnings to emphasize the negative health effects of marijuana and opioid use (ie, loss-framed) or benefits of avoiding marijuana and opioid use (ie, gain-framed) will differentially affect prevention outcomes [43,44]. Research on message framing [45-47] and prevention messages for preteens and teenagers [48-50] suggests that gain-framed messages may be effective. Specifically, research on smoking cessation messaging favors gain-framed messages about prevention and health-promoting behaviors [51-53]. For example, the communication theory indicates that gain-framed messages conveying the health benefits of quitting smoking may be optimal to promote cessation [54]. Such messages have the potential to produce behavior change through mediated pathways, such as enhancing beliefs that quitting reduces risks and reducing the attractiveness of industry branding [55,56]. Similar gain-framed messages can be adapted to marijuana and opioid use prevention to address the changing prevention environment. The use of graphic imagery has also been found to contribute to the uptake of gain-framed health messages [57,58]. Social media are effective tools to share graphic, multimedia prevention content that may enhance framing effects. LTE is promising in that it applies this approach to prevention by promoting the use of positive antidrug brand representations delivered via peer networks on social media.

### Living the Example Intervention Research

In this study, we adapted and evaluated a novel intervention, LTE, which has been previously pilot tested and demonstrated to be effective in reducing 30-day marijuana and prescription painkiller use intentions [42]. The LTE pilot was based on an after-school curriculum for *youth ambassadors*, followed by *ambassador* promotion of peer substance use avoidance with user-generated content via digital social networks and through school-wide *change projects*. LTE addresses numerous forms of substance use overall, including all widely used illicit drugs and alcohol, and the evaluation includes measures of intentions and use. This study focused specifically on marijuana and opioid use due to the rapid growth and prevalence of these substances and because the previous pilot demonstrated effectiveness in reducing use intentions.

The specific aim of this research was to implement and evaluate the adapted LTE curriculum in 3 suburban Maryland high schools. We tested 3 hypotheses:

1. LTE *youth ambassador* training would be successful in disseminating prevention messages via social media during the school year.
2. Exposure to LTE would be associated with lower 30-day substance use intentions at follow-up.
3. Higher levels of exposure to LTE would be associated with a reduction in 30-day substance use intentions across the program implementation period.

## Methods

### Design

We employed a quasi-experimental design (QED) through 3 cross-sectional surveys of freshmen at each school to test dose-response effects of exposure to LTE social media and school-based activities on substance use outcomes. We collected self-reported data on outcomes through questionnaires administered at 3 waves before, during, and at the conclusion of the program in the 3 high schools over a period of 9 months. We also collected data from the social media platforms used by students, such as Instagram, to independently measure their LTE posting activity.

### Intervention

The LTE curriculum was delivered as a series of structured after-school group sessions of 90-min each, 1 session per week. The sessions were delivered by a single program staff member following this outline:

- Session 1: What is a brand? Describe the idea of branding and branding substance use prevention.
- Session 2: Introduction to social media. Learn basics of social media, how it can influence message recipients, and how to create influential messages.
- Session 3: Boosting online engagement. How to connect and build engagement with social networks.
- Session 4: Using your voice—introduction to advocacy. Learn how to share opinions about an issue in the community with aim of influencing peers.
- Session 5: Advocacy in action. Develop skills to advocate for substance use prevention with peers.
- Session 6: Applying what we have learned. *Ambassadors* develop live content, share it with their social media networks, and engage with their peers.

Program staff, who were counselors and other school officials, recruited *youth ambassadors* at the 3 schools (46 in total), all seniors, at the start of the school year. The LTE curriculum sessions were then delivered through in-school sessions with *ambassadors* at school-assigned times. Each session was followed by staff-led questions and discussion topics to ensure *ambassador* comprehension of the content.

Following the last session, *ambassadors* were given 2 weeks to develop prevention content and share it live with their social media networks. This represented the launch of LTE prevention messaging to the broader student population beyond the *ambassador* group. After the initial *ambassador* training and launch of peer-to-peer prevention messaging, the program staff maintained weekly in-person, SMS text message, email, and instant messaging contact with *ambassadors* to encourage regular social media posting and provide help and encouragement in a coaching function. Additional activities, projects, and competitions were scheduled to keep *youth ambassadors* engaged in the LTE program throughout the course of the academic year. Competitions offered opportunities for youths to compete against each other and win prizes for their social media content and peer outreach. This strategy rewarded *ambassadors* for creative outreach techniques, effective inclusion of prevention content, persuasive and engaging tactics, compelling narrative stories, and other noteworthy features of their work. These competitions were also intended to boost intervention reach and amplify prevention messaging—for example, prizes were awarded for generating the most social media engagement (ie, shares, reactions, or comments) and using the *#livingtheexample* hashtag.

After completing the initial 6-week training curriculum, each participating school submitted a project description and budget to receive a monetary stipend to complete a “change project.” *Youth ambassadors* were challenged to design and implement change projects to increase peer knowledge and awareness about the harmful effects of substance use and address social norms, stigma, and perceptions about substance use and addiction. Approved and funded projects were implemented during the spring semester. Following successful project completion, each school submitted results of their work in the form of photos/videos and reports. These change projects were part of the overall LTE intervention and were captured in social media posts by *youth ambassadors;* measures of LTE exposure are inclusive of this activity.

In the spring of 2019, the program staff led interactive STM substance use prevention youth rallies at each participating school. Large numbers of students attended each rally, which were held as school-wide assemblies. We do not have data on exact attendance. The purpose was to draw attention to the overall substance use prevention program, generate enthusiasm surrounding the *youth ambassadors,* and stimulate interest in *ambassador* LTE social media posts*.* We note that this was a 1-time event and served to reinforce the ongoing program. The STM rally was designed to dispel marijuana and opioid use myths (ie, that most peers use marijuana or that opioid use is safe) and to encourage positive peer influence. The STM rally focused on youths’ talents and strengths, enabling them to become prevention advocates. The STM rally featured thought-provoking speakers, including *youth ambassadors*, a scientist from the National Institute on Drug Abuse (NIDA) who educated students about the science behind substance use and the adolescent brain, and a young person in recovery sharing an honest testimonial.

### Measures and Instruments

We adapted a previously tested questionnaire using validated scales from previous work by the authors [59,60], as well as from other validated scales from both the Substance Abuse and Mental Health Services Administration (SAMHSA) 2014 Communities that Care survey instrument and the 2012 Monitoring the Future survey [61]. The 88-item instrument was programmed into the SurveyMonkey software for computer-administered completion during a required freshmen English class at each of the high schools and took an average of 15 min to complete. In addition to demographic information and last grade completed in school, other scales used included the following: traditional and digital media use; attitudes toward social media; drug use risk perceptions; personal and perceived peer reasons to use drugs; drug use social norms; perceived peer drug use; reported peer drug use; self-reported past 30-day drug use; next 30-day drug use intentions; drug use/refusal influences; and self-reported exposure to the LTE program (ie, self-reported frequency of receiving social media messaging and from whom) and receptivity to the LTE program messages (ie, self-reported trust and how much they liked the program messages). The response option format for the drug use intention measure was on a 5-point Likert scale (ranging from strongly agree to strongly disagree) with statements about drug use intentions.

Additionally, we documented posts from a total of 24 (of the 46 total) *youth ambassadors* across the 3 high schools to social media platforms of their choice during an 8-month period, primarily on Instagram and Snapchat. We measured *ambassador* social media outreach activity, *ambassador* followers, type of outreach (ie, post, story, snap), and peer engagement (ie, likes, comments).

### Data Collection

Working with a local liaison at each high school, we scheduled the questionnaires to be first administered before the initiation of LTE *youth ambassador* training in November 2018 (baseline or wave 1). The required freshmen classes were invited to participate in the survey, which included a total potential sample of 923 freshmen students across the 3 high schools. We surveyed a total of 826 students at baseline (89.5% of the student census) across the 3 high schools during October and early November 2018. We did not record the students’ personally identifiable information, as the study design was cross-sectional. The training was then implemented in 6 weekly sessions followed by an *ambassador* contest to launch LTE. We completed a midline (wave 2) survey in February to March 2019 with a total of 584 students recruited from the same classes as baseline. Finally, we completed the endline (wave 3) survey in June 2019 with a total of 542 students in the same classes. Thus, at wave 3, we were able to survey 58.7% (542/923) of all freshmen and 65.6% (542/826) of those surveyed at wave 1. Ambassadors were not sampled as part of survey data collection. We cannot independently confirm whether lower rates of completed surveys at follow-up were due to student absences on the survey date or refusal to participate.

We also collected social media data from *youth ambassador* posts using Hootsuite software and captured *ambassador* posts with the included *#livingtheexample* hashtag. Owing to the nature of the Instagram platform, stories are only available for a 24-hour period; in some cases, program staff requested that *ambassadors* provide screenshots of their posts for documentation purposes.

### Data Analysis

Drug use intentions, LTE exposure, and demographic (age and gender) data from waves 2 and 3 were appended into a single dataset. We first estimated ordinal logistic regression models for each drug use intention variable in wave 3 (ie, sell/distribute illegal drugs, smoke cigarettes, drink beer/wine/hard liquor when parents do not know about it, use marijuana, use lysergic acid diethylamide (LSD)/cocaine/amphetamines or other illegal drugs, use heroin, use synthetic drugs, use any prescription pills without a prescription) to examine the association between LTE exposure and drug use intentions. We assumed that the relationship between each pair of outcome categories was the same, meaning that the coefficients that describe the relationship between strongly agree versus all higher categories of the response are the same as those that agree versus all higher categories and so on.

In a separate set of regressions, we included an interaction term for the study wave to examine whether participants had stronger disagreement with drug use after the intervention was implemented. All regression models controlled for participant’s age and gender. All data analysis was conducted in R version 3.5.3 (R Core Team, 2019). All regressions fit model assumptions have been described.

## Results

We recorded LTE social media activity from a total of 24 (of the 46) *youth ambassadors*. The Instagram story function was the most widely used by *ambassadors*, with a total of 45 stories, followed by 28 Instagram posts made during the program. The *ambassadors* had a total of 12,894 followers in their social media networks during the program period. There were 1291 follower likes for posts by *ambassadors* and 33 comments made by followers, likely a partial representation of engagement. It is worth noting that we cannot confirm that we captured all social media posts created by the *ambassadors* as some students may not have used the hashtag or provided complete data to the coordinator on their posting activity. It should also be noted that, due to the nature of social media platforms, we cannot confirm the identity of individuals in the *youth ambassadors’* networks who engaged with the posts or how many of them represent unique individuals.

In the outcome surveys, we collected limited demographic information, and 52% of respondents were female, 55% were aged 14 years and 45% were aged 15 years, 96% were born in the United States, and all were freshmen. The survey focused mainly on substance use attitudes, beliefs, intentions, and behavior. Mean responses to the drug use intention scales for the outcomes of interest are shown in Table 1.

**Table 1:** Overall mean differences in drug use intentions among all baseline, midline, and endline groups.

Measures	Baseline (n=835), mean (SD)	Midline (n=593), mean (SD)	Endline (n=557), mean (SD)	*P* value
Sell/distribute illegal drugs^a^	3.69 (0.68)	3.58 (0.82)	3.54 (0.83)	<.001
Smoke cigarettes	3.72 (0.60)	3.67 (0.69)	3.57 (0.81)	<.001
Drink beer, wine, or hard liquor (eg, vodka, whiskey, or gin) when my parents do not know about it	3.56 (0.77)	3.47 (0.85)	3.36 (0.93)	<.001
Use marijuana	3.56 (0.81)	3.48 (0.88)	3.36 (0.99)	<.001
Use LSD, cocaine, amphetamines, or other illegal drugs	3.74 (0.62)	3.72 (0.65)	3.63 (0.77)	.008
Use heroin	3.75 (0.61)	3.72 (0.66)	3.65 (0.77)	.013
Use synthetic drugs (eg, K2, spice, bath salts)	3.75 (0.60)	3.74 (0.64)	3.63 (0.77)	.002
Use prescription pills without a prescription	3.71 (0.65)	3.66 (0.73)	3.59 (0.80)	.007

^a^For each of the following questions, please indicate whether you strongly agree=1, agree=2, neither agree nor disagree=3, disagree=4, or strongly disagree=5.

Next, we estimated ordinal logistic regression models for each of the 30-day substance use intention outcomes of interest (Table 2). In this model, we found a significant positive effect of LTE exposure on all 8 measured drug use intention variables: sell/distribute illegal drugs; smoke cigarettes; drink beer, wine or liquor when my parents do not know about it; use marijuana; use LSD, cocaine, amphetamines, or other illegal drug; use heroin; use synthetic drugs; use any prescription pills without a prescription (all *P*<.05; odds ratios ranging from 2.12 to 3.71). We also found that boys were more likely than girls to exhibit reductions in drug use intentions.

**Table 2:** Logistic regressions of drug use intentions on Living the Example program exposure at wave 3, exponentiated coefficient (n=542).

Measures	Self-reported drug use intention, exponentiated coefficient (*P* value)							
	1^a^	2^b^	3^c^	4^d^	5^e^	6^f^	7^g^	8^h^
LTE^i^ exposure	2.96 (<.003)	3.42 (<.001)	2.12 (<.007)	2.91 (<.003)	3.61 (<.001)	3.58 (<.001)	3.71 (<.001)	3.44 (<.001)
Age (years)	0.99 (.94)	0.85 (.33)	1.08 (.56)	1.23 (.15)	0.94 (.73)	0.81 (.23)	0.93 (.64)	0.98 (.90)
Gender (female; reference)	2.72 (<.006)	2.71 (<.007)	1.94 (<.009)	2.74 (<.006)	3.60 (<.001)	3.24 (<.003)	3.03 (<.004)	2.76 (<.006)

^a^Sell/distribute illegal drugs.

^b^Smoke cigarettes.

^c^Drink beer, wine, or hard liquor (eg, vodka, whiskey, or gin) when my parents do not know about it.

^d^Use marijuana.

^e^Use LSD, cocaine, amphetamines, or other illegal drugs.

^f^Use heroin.

^g^Use synthetic drugs (eg, K2, spice, bath salts).

^h^Use any prescription pills without a prescription.

^i^LTE: Living the Example.

Next, we estimated a separate model that included a survey wave interaction term to examine the hypothesis that substance use intentions declined during the LTE program as a function of exposure to peer-to-peer prevention messages via social media. As hypothesized, we observed reductions in 30-day intentions to use all 8 measured drugs between the second and third follow-up surveys, the period during which the social media messaging component of the LTE program took place (Table 3).

**Table 3:** Logistic regressions of drug use intentions on Living the Example program exposure and survey waves 2 to 3, exponentiated coefficient.

Measures	Self-reported drug use intention, exponentiated coefficient (*P* value)							
	1^a^	2^b^	3^c^	4^d^	5^e^	6^f^	7^g^	8^h^
LTE^i^ exposure	3.31 (<.001)	3.30 (<.001)	2.43 (<.008)	2.58 (<.007)	3.33 (<.001)	3.73 (<.001)	3.57 (<.001)	3.45 (<.001)
Wave 2 (reference)	1.06 (.66)	1.29 (.07)	1.16 (.24)	1.14 (.32)	1.30 (.09)	1.21 (.21)	1.41 (.02)	1.16 (.31)
Age (years)	1.21 (.08)	0.99 (.91)	1.15 (.17)	1.31 (.01)	0.98 (.89)	0.98 (.85)	1.03 (.83)	1.13 (.31)
Gender (female; reference)	2.35 (<.009)	2.00 (<.012)	1.48 (<.018)	2.15 (<.011)	2.42 (<.007)	2.43 (<.007)	2.16 (<.011)	2.11 (<.011)

^a^Sell/distribute illegal drugs.

^b^Smoke cigarettes.

^c^Drink beer, wine, or hard liquor (eg, vodka, whiskey, or gin) when my parents do not know about it.

^d^Use marijuana.

^e^Use LSD, cocaine, amphetamines, or other illegal drugs.

^f^Use heroin.

^g^Use synthetic drugs (eg, K2, spice, bath salts).

^h^Use any prescription pills without a prescription.

^i^LTE: Living the Example.

## Discussion

### Principal Findings

LTE had a positive effect on reducing substance use intentions among high school students. Overall, results of this study were consistent with and indicate a stronger LTE effect in this study compared with the 2016-2017 previous pilot study [42]. LTE appears to offer a protective effect, with exposure to program messages leading to reduced drug use intentions. This approach deserves further development, refinement, and evaluation using rigorous randomized controlled methods that capture detailed dose-response data.

With regard to our hypotheses, first we saw some evidence that *ambassadors* were successful in reaching their peers. We were able to independently record social media posts for 24 *ambassadors* who produced LTE messages and shared them with nearly 13,000 followers over a period of 8 months. This demonstrates the feasibility of the LTE model reaching a large number of youths using a peer-to-peer approach at a low cost. Although the LTE program intended to leverage social media platforms that were most widely used by youth participants and their peer networks, some platforms, including Instagram, presented unique challenges for program implementers and evaluators. Instagram stories disappear from a user’s profile after 24 hours, introducing barriers for documenting the content, features, and persuasive tactics used by *ambassadors*. Additionally, some ambassadors had private Instagram profiles, which prevented our documenting their posts. The abbreviated postduration also limits the extent to which the content can be viewed by followers and potentially shared on follower profiles. Regardless, LTE demonstrated significant potential to reach large numbers of peers via social media networks, with some months having over 10,000 followers of *ambassadors* on Instagram when there were only 14 *ambassadors* making at least one post during that period.

Second, we found that exposure to LTE is indeed associated with reduced 30-day drug use intentions, confirming our previous pilot study findings [42]. In our first set of regressions, we found that self-reported exposure to LTE was associated with lower use intentions across all 8 measured substances, including widely used drugs such as marijuana and prescription painkillers (opioids). This suggests that LTE is an effective approach that deserves large-scale implementation and evaluation.

Third, we found evidence of a dose-response relationship between LTE exposure and reduced 30-day drug use intentions. There was an effect of exposure to peer-to-peer messaging over time, with youths who self-reported exposure showing reduced use intentions at the wave 3 endline follow-up. Dose-response effects are important evidence for the effectiveness of media interventions, first as they demonstrate a direct relationship between the media stimulus and outcomes of interest and second as they provide evidence for the magnitude of those effects [62]. Optimizing dosage is an important objective for health campaigns [63] and for this research.

### Future Research and Limitations

Future research should use a randomized controlled trial design to compare implementing and nonimplementing schools and examine dose-response effects in detail. A group randomized trial involving sufficient numbers of schools for statistical power purposes assigned both to implement and not implement LTE across a school year would provide confirmatory evidence of the program’s effectiveness. Together with detailed measurement of social media activity both through independent monitoring of posting activity at the individual *ambassador* level and through self-report, a future study such as this could specify the optimal dosage for LTE to produce meaningful prevention effects.

There are some limitations to this study. First, the number of survey respondents declined in wave 2 and 3 follow-ups, but we cannot confirm whether students were not in attendance during survey administration or they did not agree to complete the follow-up surveys. Second, we were only able to independently confirm LTE social media activity for 24 of the 46 *ambassadors.* We were unable to track potential posting for some *ambassadors* due to the omission of program hashtags in posts, due to use of the hashtags but with private Instagram account settings, or due to posting on the Snapchat platform, which does not permit hashtag tracking and is otherwise quite difficult to monitor. Additionally, we cannot quantify how many of the posts we documented, and engagement statistics we captured, represent unique individual users. Consequently, our independent social media data are incomplete. Finally, due to limitations with the social media monitoring, we were not able to statistically analyze the relationship between independent social media metrics and outcomes measured in the surveys.

### Conclusions

Adolescent substance use continues to be a pressing public health threat in the United States, in particular due to the growing availability of lethal drugs, teen social norms shifting to be more favorable of recreational use, and growing perceptions of the relative safety of some substances [2-4]. Digital social networks are major drivers of public perceptions of substance use [7,25,26], calling for novel digital strategies for prevention in this space. LTE offers a promising model for future programming seeking to widely disseminate peer-to-peer prevention messaging via social media to reduce adolescent drug use intentions. By training a relatively small number of youths, there is potential to reach a large audience with authentic, targeted prevention messages, given the widespread use of social networking platforms. Future research should build on the evidence of the effectiveness of LTE by implementing and evaluating the program at a larger scale using randomized controlled methods.

## Abbreviations

GW: George Washington
LSD: lysergic acid diethylamide
LTE: Living the Example
NIDA: National Institute on Drug Abuse
PYD: Positive Youth Development
QED: quasi-experimental design
SAMHSA: Substance Abuse and Mental Health Services Administration
STM: Shatter the Myths
